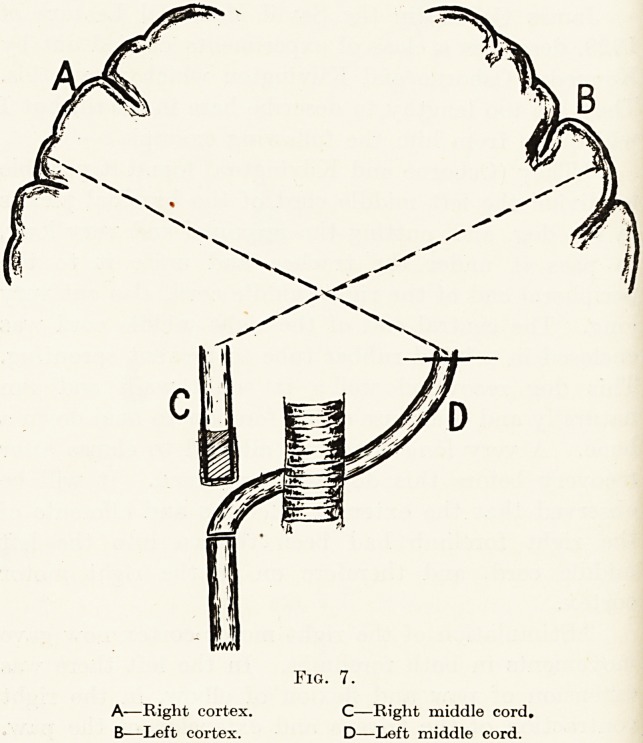# Some Observations on the Relationship between Structure and Function

**Published:** 1932

**Authors:** J. R. Charles

**Affiliations:** Lecturer in Medicine, University of Bristol; Honarary Physician, Bristol Royal Infirmary


### The Bristol Medico-Chirurgical Journal

*"Scire est nescire, nisi id me*
*Scire alius sciret."*

SPRING, 1932.

### SOME OBSERVATIONS ON THE RELATIONSHIP BETWEEN STRUCTURE AND FUNCTION.

The Presidential Address, delivered on 14th October, 1931, at the opening of the Fifty-ninth Session of the Bristol Medico-Chirurgical Society.

BY

J. R. Charles, M.A., M.D., F.R.C.P.,

*Lecturer in Medicine, University of Bristol;*
*Honorary Physician, Bristol Royal Infirmary.*

"Structure is only the intimate expression of function. . . ."—*John Hunter.*

"Function implies organization adapted to the attainment of a goal."—*McDougall.*

It has recently been said by Sir Arthur Thomson that man's body is a walking museum of relics. Being the heir of all the ages, man has inherited structures which have become atrophied because they no longer subserve any purposive functional requirement, and persist only as interesting and all too often noxious remnants.

MAY 6 1932

While this is undoubtedly true, there is another and brighter side to the picture—brighter, that is, if we realize that it is functional requirement which determines structure, and uses this as its handmaid. For if this be true, man holds his future evolutionary destiny very largely in his own hands; but that future must be determined by individual effort and conscious striving, which is all-important because it leads to differentiation, lifting mankind above the level of the herd to greater heights.

The purpose, then, of this address is to support the view that Function is the cause of Structure. In our medical work we are so apt to have an eye on structure, with the subsequent derangement of function which must inevitably ensue if structure be deficient or defective, that we are very liable to fall into the error of supposing, or tacitly assuming, structure to be the all-important thing. It is our business in life for the main part to study disordered structure, and so I think there is a tendency for us to fall into a frame of mind which lays far too much stress on structure, both normal and morbid.

Structure does not exist for itself, it merely exists to subserve the needs of function. We are liable to err so greatly in this direction as to think that function can spring from structure; in fact, we go as far as to say that this or that structure, be it the heart, a gland, a muscle, bone or even nerve cell, has such or such a function, and therefore, surely, we develop in our own minds a fixed idea, which is very largely false. It appears to me that until we train our minds to appreciate our wrong attitude, and to bear always in mind the idea that functional requirements precede structure, we shall not attain anything approaching complete success in the treatment of human beings who are suffering from what we call a disease.

We fix our minds on structures, cut sections, make pictures and think we understand the trouble. This is perhaps all the more fallacious because the method yields a half truth, and leads to fatal contentment. I do not wish to discredit the work of morbid anatomists; rather do I mean that we must ever regard structure merely as a something through which function expresses itself, and not confuse it with function itself. This is fatally easy in all forms of so-called organic disease, but perhaps most particularly so in diseases of the nervous system, especially in diseases of the mind.

It is curious that the "structural view" is so prevalent; we might with equal validity assert a loud speaker to be the cause of wireless waves, or an electric vacuum bulb the cause of light. We know well enough that such statements would be absurd, and that if we damage our loud speaker we hear no sound, and if we break our bulb we have no light. Yet each is only a mechanical means of expression of ulterior forces or waves, *i.e.* they are physical structures through which certain functions are displayed.

It is doubtful whether we are right in classifying diseases into organic and functional groups as we do to-day. Every disease and symptom is functional, though some are associated with visible changes in structure; all disease is a disharmony or perversion of function. By regarding disease in this way we shall, by studying these disordered functions, and having a fuller knowledge of physiological processes, be able to assist nature by means of (*a*) re-education, (*b*) compensation, (*c*) acclimatization, (*d*) adaptation, etc.

What are some of the grounds on which I base my opinion that functional requirements determine structure ? I think the first and most fundamental evidence with regard to this view must be sought in the evolution of mankind, and as it would clearly be impossible to trace much of this in any detail, I propose limiting my remarks almost entirely to certain aspects of the nervous system ; but before doing so I will briefly digress to give an example from a recent book of J. S. Haldane, *The Philosophical Basis of Biology.* In a chapter on breathing he describes acclimatization to high altitudes, and writes :—

" For one thing, as has been known for long, the blood becomes richer in hæmoglobin. This does not, by itself, raise the oxygen pressure in the arterial blood ; but with the same rate of circulation it lessens the fall in oxygen pressure as the blood passes through the tissues, and thus tends to raise the oxygen pressure in them, which is the essential matter. The percentage of hæmoglobin in the blood has recently been shown to vary inversely, not only with the oxygen pressure when this is below normal, as at high altitudes, but also when it is above normal, as when an animal is kept at a high atmospheric pressure, or in air enriched with oxygen. This illustrates very clearly the connection between function and structure. The bone marrow, where the red corpuscles of the blood are produced, becomes altered in structure at a high altitude, just as the blood itself is altered in structure."

It seems to me that we can trace a something, call it what we will (? wisdom of the body), which has been a continual striving towards a further end, *i.e.* something which is essentially teleological in quality and which still manifests itself in the highest achievement of the body of man, namely " the mind." Moreover, with complexity of function comes complexity of structure. It is a platitude to say that without bones in a leg there would be no support, or that if any other important structure in the body goes wrong its function is proportionately altered. But this is merely because the said structure is the means whereby the said function is exhibited, and is not itself the function.

As we trace the development of the nervous system from its most lowly beginnings we find that the nerve cells are essentially cells which have wandered from the sensory surface which is in communication with the outside world, and form connecting-links between the outer surface and the inner motor cells ; *i.e.* the nervous system is always in essence a collection of intercalated cells. In man the "neural groove" is formed by a process of tucking in a strip of the skin of the back ; the edges of this groove meet, form a tube, sink into the body and eventually form the whole of the central nervous system, brain, cord and nerves. A cell at the edge of the groove may happen to remain behind, and so remain a skin cell, or it may be included in the neural groove and become a nerve cell. Therefore, for a given cell it is a matter of mere chance whether it becomes a structure with the functions of a nerve cell or a structure with the functions of the skin. The cells which are certain to be included in the formation of the groove, viz. those which lie in the mid-line, go in first and deepest, thus forming the motor cells of the cord, while those which turn in last (some of which, as we have just seen, may form nerve cells or may remain as skin cells) are intimately related to the skin, and thus form the posterior part or sensory aspect of the cord. (Fig. 1.)

We see the same arrangement even in the floor of the fourth ventricle, where the cells nearest the mid-line are motor in function, those of the sides sensory in function. In this arrangement, then, it is seen that it is function which has determined structure, and not *vice versa*.

Moreover, we see that there may be some justification for considering the skin and sense organs

FIG. 1.

almost as an external nervous system, viz. a part of the body in communication with the outside world. Thus we must ever bear in mind the very intimate relations between the functions of the skin and those of the nervous system which is derived from it.

The external environment affects the external cells of the body from which we have seen the nervous system is derived. Various environmental stimuli impinge on the external integument (and sense organs), and as the nervous system is developed evolutionarily, structure is found to accommodate and utilize the various functional impressions which fall upon it.

I think we are quite justified in using the word "functional" for these impressions, for differences in temperature, pressure, touch, sight, hearing and smell may all be resolved into molecular bombardment or waves of something—air or ether. It is the business of external cells to be influenced by the environment, and the business of internal cells to respond to the functional waves conducted from the external. With a change in environment the functional requirements may produce a rapid change, even in the appearance (*i.e.* structure) of the epidermis (external integument), as in such animals as fish, caterpillars, etc. An octopus, for example, may settle on a rock, and then change its colour in harmony with that of the rock.

The animal, therefore, becomes by functional requirements adapted to its surroundings for self-protective or combative purposes, and its behaviour may be modified accordingly. Hence there is an intimate connection between (1) external environment—waves of many varieties, (2) skin and special senses,

(3) nervous system and behaviour ; and it is function which, originating in (1), passes through (2) and partly determines (3).

When the structure has finally been produced it is so built that it serves a particular function ; but many examples could be quoted where a structure which has what may be described as a given function may quite easily be made artificially to subserve a different or even opposite function (if so required), *e.g.* a flexor muscle of the forearm may be readily converted into an extensor by surgical manipulation.

Quite early in the embryonic development of the brain groups of nerve cells are developed which have sensory functions for sight, hearing and smell. These are known as placodes. With regard to the visual placodes, these are formed before the neural tube of the brain is closed. When, therefore, this closure occurs the visual placodes are turned inwards. The structures may be perfect enough, but it is clear that if they remained in this position they would be quite useless functionally for external vision. But vision is their function, and the functional requirements therefore demand a very different structural arrangement, and succeed in obtaining their purpose. This they do by growing outwards till they meet the skin, which, however, is semi-opaque, and the requirements of function demand that this semi-opacity should be altered, and therefore the appropriate cells of the skin become transparent to form the cornea and lens. (Fig. 2.)

That some extraordinary influence is exerted with a purpose in view is seen by the fact that if a tadpole's developing eye be grafted under the skin of different parts of its body, *e.g.* the abdomen, this same purpose is responded to, and a lens formed from the abdominal skin. The human eye, however, as it grows outwards is still inverted, and this explains why in man the retinal nerves lie in the inner side of the visual cells. (Figs. 3 and 4.) Some unknown purpose has enabled it to attain its object, in spite of this structural imperfection. When it is fully developed it can, however, only appreciate the ether-borne waves of light—wave lengths from 400 to 800 millimicrons. In fact, its function is after all a very limited one, like that of the ear, which only appreciates air-borne waves within the short range of frequency of 40

*By courtesy of Messrs. Edward Arnold & Co.*

FIG. 2.

Development of vertebrate eye.

a second to 4,700 a second. And yet these functions of sight and hearing, combined with the other special senses, and the sensory functions of the skin, dominate the motor system as with a purpose. For we find motor cells in close proximity to and linked up with the synapsis which transmits the sensory impressions, motor cells which are capable of carrying out the appropriate motor responses. We recognize a similar arrangement in the cord, and we see the same in connection with vision, and observe how the third, fourth and sixth nuclei are joined by the posterior longitudinal bundle, to carry out the necessary movements of the eyes.

Thus we have reached a stage of embryonic development which has innumerable and appropriate reflex responses. The pathways of these are laid down in the embryo before they are used, and they are laid down presumably as the result of racial memory. But more than this more or less automatic responsive mechanism was needed if racial progress was to be made. There appears to have been evolved a purposive effort to control movement and adapt it to fresh and unusual circumstances.

Thus new function required a fresh structure through which it could act, and this fresh structure consisted of an ever increasing and complex mass of cells, intercalated between the incoming sensory impressions and the outgoing motor responses. This new mass of cells was the pallium, which was not developed in the midst of the already existing basal ganglia, but took the form of hollow bilateral outgrowths from the brain stem. With the rise of the pallium we reach a stage of individuality, and individuality implies retention of acquired memories, conscious control, etc.

*By courtesy of Messrs. Edward Arnold & Co.*

Fig. 3.

Inverted eye of a vertebrate.

*By courtesy of Messrs. Edward Arnold & Co.*

Fig. 4.

Eye of an invertebrate.

Now concerning the volume of the brain in relation to function, it cannot be said that the larger the structure the larger the function. A whale, for instance, possesses the largest brain (about ten pounds), but a very small one in comparison with the bulk of its body, the ratio of brain to body weight being 1—25,000. The weight of a human brain is about 48 ounces, and its ratio to body weight about 1 to 60.

The convolutions of the brain are produced by the development of the cortex requiring a larger surface area, but it must not be assumed that multiplicity of convolutions *per se* constitutes any indication either of intelligence or increased function. The whale has the most convoluted brain of all brains, but a much more intelligent small mammal has a smooth, more or less unconvoluted cortex. This question of convolutions, other things being equal, seems to be dependent on the size of the animal, and the relation of the size of its brain to its body mass. The brain in an animal may be relatively a thousand times larger than that of the whale in proportion to body mass, and yet be quite smooth on the surface.

Although there has been a great deal of dispute over the question, it appears now to be conceded that the disposition of the convolutions is not in any sense haphazard, and that they are not merely formed because of the relative smallness of the cranial vault, but that the different convolutions have different cell structures, some forty of which have been recognized.

This, I think, is the important point, that these different structures have different functions, and that it is the functions which have determined the structures, the pallial structure being the outcome of bodily functional requirements. Structure here is the expression of function, and we must beware of the fallacy of regarding the cortex as a mosaic of rigidly delimited functional areas. As the pallium develops the olfactory area becomes relegated to a lower position, while areas associated with more important functions are more fully represented in the cortex ; yet most of us know how potent a smell may be in recalling some event which was associated with a similar odour.

The final stage is the development of neutral areas, which form the association areas so vastly important in the brain of man, which distinguish it from those of lower animals. The function of the brain of man is so far removed from that of even the higher animals, that the results of experiments carried out on their brains cannot be accepted as being applicable to man, except with the greatest caution. The human brain differs from that of the higher animals not so much in the number of cells which it possesses, that is, of the grey matter, but in the very much greater amount of white matter (*i.e.* association fibres) weight for weight. It is for this reason a much better organ functionally, but it appears to be more dependent on solitary tracts, and therefore certain local lesions may produce permanent effects.

There are a few points in connection with the cortical representation of movement which, perhaps, we are apt to overlook. The face, which from an evolutionary point of view is all-important, has its position at the lower end of the precentral cortex. Immediately above that we have the centre for the hand, then forearm, arm, shoulder, trunk, hips, thighs, legs, toes. (Fig. 5.) This, it will be observed, is not the anatomical order, which we might expect would run —face, shoulder, arm, forearm, hand, trunk, and so on. What is the reason why the order of the representation of the parts of the upper arm has been reversed? Almost certainly because the development of the important function of the hand during evolution followed the importance of the face, and therefore took up a structural position close to it. Here, surely, is a clear example of function determining structure. This arrangement is peculiar to the cortex, for as the fibres from these cells descend, forming the pyramidal tracts, a twist occurs, so that at the internal capsule the order from before backwards follows the anatomical order, and not that which we observe in the cortex, *i.e.* the fibres run in the order of the face, shoulder, arm, forearm, etc. Further, there is one other point to be observed. These fibres arise from the Betz cells in the cortex; those for the face, which are lower

FIG. 5.

in the cortex, have a short distance to travel before they form their synapsis at the facial nuclei. Those for the legs have a long distance to travel, from the top of the cortex to the lumbar area. We find in accordance with this that the Betz cells which subserve the nutrition of the leg fibres are very much larger than those which subserve the nutrition of the face fibres, with a gradual diminution in size of the Betz cells of the cortex as we pass from leg to face area. (Fig. 6.) Here is another example of increased requirements of function receiving greater structural mass. It will be agreed that the functional capacity of a hand and arm is much greater than that of a leg; yet the Betz cells which are concerned with the mechanism of movement of the hand and arm are smaller than these corresponding cells for the leg. This again indicates that it is not so much the cells as the functional associational pathways which are of even more importance than the cells.

It might be inferred from such observations that there are definite localized motor areas in the brain, for face, arm, leg and so on, from which impulses arise causing movements in these different parts. This view, which was at one time held, cannot be sustained any longer. Accumulating evidence appears to prove that the functions of the brain are very much more widespread throughout its structure than was formerly supposed, and that it tends to function much more as a whole than was thought, and probably the so-called localizing areas are not spots where movements or feeling are initiated, but rather areas which merely subserve the transmission of impulses concerned with these special movements or sensations.

We must not suppose that a given function resides in a given structure, *e.g.* that a cortical motor arm area

FIG. 6.
Betz cells.

can only subserve movements in the contralateral arm; that that is the normal course of events all will be agreed, but the requirements of the body may force this structure to take up other functions. A large number of most interesting experiments have been carried out which bear on this question, which seem to point to the view that open pathways are more important than localized areas. In other words, here again structure will be subservient to function. A given area of the cortex may even be made to assume additional functions.

James Collier, in the Savill Memorial Lecture of 1929, describes a class of experiments carried out by Kennedy, Osborne and Kilvington which prove this. They are too lengthy to describe here in detail, but I will quote from him the following example:—

"They (Osborne and Kilvington) found it possible to divide the left middle cord of the brachial plexus in the dog, and, cutting the proximal end very long, to pass it under the trachea and unite it to the peripheral end of the right middle cord, also cut very long. The central end of the right middle cord was enclosed in a blind rubber tube to prevent sprouting. This dog recovered well; it could walk and run naturally and could use either forepaw to hold down a bone. A very long time was allowed to elapse after recovery before this dog was examined. It will be observed that the extensor afferents and efferents of the right forelimb had been thrown into the left middle cord, and therefore on to the right motor cortex.

"Stimulation of the right motor cortex now gave movements in both forelimbs. In the left there was extension of paw and flexion of elbow, in the right contraction of the triceps and extension of the paw.

Stimulation of the left cortex produced movement in the right limb only, entirely excluding any movement in the triceps or paw extensors. To put it shortly, the cortical points for extension of the right forelimb of this dog had been removed from his left hemisphere, and had been implanted in his right hemisphere. The left middle cord was then divided high up, and its distal end stimulated, when exactly the same movements occurred as had resulted from stimulation of the right cortex." (Fig. 7.)

FIG. 7.

A—Right cortex. C—Right middle cord.
B—Left cortex. D—Left middle cord.

Certain rules are therefore formulated :—

1. " If a peripheral nerve is cut the corresponding motor cortex loses its electrical excitability. (This, I [James Collier] submit, is only another way of saying that if you block an efferent path near its periphery there can be no result from stimulating that same isolated path, as it leaves the cortex, and I infer from this that the results of stimulation in the motor area are those of stimulation simply of an outgoing path and not stimulation of a centre of function.) "

2. " It does not matter to which area of the motor cortex, nor to which hemisphere you transfer your peripheral innervation ; complete recovery of function occurs — motion, co - ordination and sensibility — provided the path in and the path out are fully restored. (From which I infer that the path is much more important than the whereabouts in the cortex.) "

3. " In this recovery of function the afferents must be all important."

For without the different proprioceptive impulses perfectly co-ordinated movements would not be " trained into " the parts of the cortex which take up their fresh functions.

The brain is a structure into which myriads of impulses, silent (proprioceptive), distant (exteroceptive), visceral, conscious and unconscious are perpetually pouring from every direction, there to be transformed at different levels into reflex actions, cardiac and respiratory movements and volition. While the lower centres are fixed, the upper are much more mobile than was at one time supposed. The pathways along which these impulses can run are all important, and are composed of synapses and association fibres which form the bulk of the brain structure. Here function is transmitted, but neither the structural pathways nor the cells are function itself.

I have just said that the lower centres are fixed, but even at this low level do we not daily see an example of altered function, while the structure of the reflex arc itself remains intact, in the extensor response of the great toe when pallial control is cut off ? Is not this an exhibition of atavistic reversion, which is seen also in the babe before the pallial fibres are myelinated ?

If, then, we can so regard the functions of the brain in relation to such relatively gross properties as movement, sensation, equilibrium, vision, and so forth, we shall not be content without a few inquiries with regard to the relation of the structure of the brain to the mind. It seems clear that the brains of many aments are defective in the number and development of their cortical cells, especially in the pyramidal and fusiform layers. The association systems also show a very definite diminution.

Various observers have also attempted to show that different mental disturbances are associated with structural alterations in cells at various levels in the cortex. But does this mean that different mental traits or characteristics can be bottled up, as it were, in different cortical cells ? The idea savours too much of the phrenologists.

I have recently read that " cellular studies of the cortex suggest that there are present richly receptive focal points of intelligence." This sort of remark implies that intelligence can or will be resolved into a question of chemistry and physics, and that intelligence has a physical basis. Let us hear, therefore, what such a distinguished physicist as

Sir A. S. Eddington has to say with regard to such a view-point :—

"Starting from æther, electrons and other physical machinery, we cannot reach conscious man and render count of what is apprehended in his consciousness. Conceivably we might reach a human machine interacting by reflexes with its environment ; but we cannot reach rational man morally responsible to pursue the truth as to æther and electrons. . . . We have followed the latest developments of relativity and quantum theories because they contain the conceptions of modern science. . . .

"What, then, is the physical basis of nonsense ? The problem of nonsense touches the scientist more nearly than any other moral problem. If the brain contains a physical basis for the nonsense which it thinks, this must be some kind of configuration of the entities of physics — not precisely a chemical secretion, but not essentially different from that kind of product.

"It is as though when my brain says seven times eight are fifty-six its machinery is manufacturing sugar, but when it says seven times eight are sixty-five the machinery has gone wrong and produced chalk. But who says the machinery has gone wrong ? As a physical machine the brain has acted according to the unbreakable laws of physics ; so why stigmatize its action ?

"This discrimination of chemical products as good or evil has no parallel in chemistry. We cannot assimilate laws of thought to natural laws ; they are laws which *ought* to be obeyed, not laws which *must* be obeyed ; and the physicist must accept laws of thought before he accepts natural law.

"*Ought* takes us outside chemistry and physics.

It concerns something that *wants* or esteems sugar, not chalk, sense or nonsense.

"A physical machine cannot *esteem* or *want* anything; whatever is fed into it it will chaw up according to the laws of its physical machinery. That which in the physical world shadows the nonsense in the mind affords no ground for its condemnation. In a world of æther and electrons we might, perhaps, encounter *nonsense;* we could not encounter *damned nonsense.*"

At any rate, there is a school of thought which lays the greatest emphasis on structure (cortical architecture) in relation to disease of the mind. But, excluding amentia and dementia, most of the upsets of mentality as seen by the neurologist are associated not so much with changes in the cortical cells as with lesions in the association systems. It seems fair to say that this school holds an essentially mechanistic view of the mind, reducing it to an extremely complicated form of machine.

Mental life, they say, is entirely dependent on the mechanistic processes of our brains and bodies. There appears to be little doubt that the state of mind influences the processes of the body. The bodily effects of fear and other emotions are well known, and undoubtedly structural changes in vital organs frequently follow prolonged and intense mental strain, anxiety, etc. Conversely, chemical alterations in the blood, due to excess and diminution of internal secretions, gout and other metabolic disorders, disturbances of the liver, stomach and so forth, do, we know quite well, alter the mentality of the patient.

The mind, therefore, may act on and be acted on by almost every part of the body—but this does not tell us what mind is, or whether or not it can utilize the structure of the body with a purpose in view, *i.e.* teleologically. If it can do so surely the purely mechanistic or structural view breaks down.

Now man can form an ideal, work for an object, strive towards it, and modify the means he adopts to attain his end. On the intellectual side he has faculties of reasoning, judgment, perception, imagination and so forth, all of which tend to produce action. Put in another way, these are functional factors which act as the exciting causes of action, because "function implies organization adapted to the attainment of a goal."

As McDougall says, "The essence of our mental life, in its aspect as intelligence, is not a mere succession of sensations, ideas or representation of things, but a *thinking* of things and of their spatial, temporal and causal relations, and such *awareness* of relation plays an essential part in the guidance of our actions."

The striving towards an end or goal is purposive, and the more obstacles encountered in the way, so much the greater is the energy which is exerted to attain the desired end. Moreover, there are four characteristics of such striving: (*a*) adaptability, (*b*) persistence, (*c*) increase of energy in proportion to the resistance offered, and (*d*) cessation of energy when the goal is attained.

These imply foresight, forward temporal reference, spatial relations and causal relations, properties which are completely at variance with those of any machine.

The function of memory is the determination of the course of present action in accordance with past events. Mind itself has been described by those holding a mechanistic view as the working of associative memory, and all mental activity, under this view, is determined by the material structure of the brain. However, the facts of memory do imply that past events have left some mark upon the organism, which endures and plays a part in settling present action. But it also plays its part in guiding this present action with regard to the future; in fact, the primary function of memory is that it may be used for anticipation, or the striving towards some goal, which is the primary characteristic of all intelligent purposive action. So that although memory is determined by the past, it works towards the future, and in this striving "becomes Imagination, the function in which mind manifests most clearly its creative power."

Here we must consider a few points in connection with the basis of memory. What is the nature of the "traces" or marks upon the organism which have been left by the function of memory? Biological mechanists say the organization of the living creature is altogether material, and that nothing counts but matter and its spatial arrangement. Therefore, according to them a desire, an intention, or a sorrow must be in some way material. They also hold that there is always material continuity of vital organization, derangement of the material structure always resulting in disturbance or abolition of all evidence of organization. They state that function is correlated to material structure. This may be true, but can only be wholly true if the correlation be perfect. If it can be proved to be perfect, then function is entirely determined by structure.

Now, it has been shown that the spatial arrangements of a developing embryo may be grossly distorted, and nevertheless later on may be rectified and return completely to normal. Therefore there must be something more than the material structure to produce this result. Similarly the leg of a newt may be cut off at any point, and will then completely regenerate. Where is the structure which produces this function of regeneration? Moreover, another limb may be cut off while the former is regenerating, without in any way interfering with the regeneration of the former, and will itself be replaced by a perfect limb.

With regard to the effect on the mind, especially on memory, of lesions in the structure of the brain, no exact localization of mental functions has been discovered. Furthermore, in connection with memory one may hear a speech, which is not, however, recorded in the structure of the brain, as it might be on a gramophone record; no single sentence of the speech may be able to be recalled, but the speech may be fully remembered, because the exact meaning of it was appreciated. Our present knowledge could not explain this in terms of structure.

The correlation between brain structure and mental processes has not been established. More and more it appears that the brain functions as a whole, and as we have seen, impaired functions may be restored and become correlated to parts of the brain other than those with which they were formerly correlated. Consider a case of complete hemiplegia, due to a complete destruction of an internal capsule by hæmorrhage. We know that such a patient may be almost completely restored functionally, even though his cortex on the affected side has been entirely and permanently cut off by the lesion. His functional recovery must mean that some other part, presumably the other cortex, has adopted fresh functions.

Moreover, the patient can walk, *i.e.* the two legs do not move synchronously but alternately. Therefore, presumably the homolateral cortex has not only taken on an increased function, but an entirely new one. The function therefore showed a relative independence of its structure. Whatever function may be, its nature is such that it cannot be revealed to us in the form of structure.

There is a strong analogy between *heredity* and *memory*, but neither the one nor the other has, as yet, been explained on solely structural grounds. McDougall says that neither the normal processes of morphogenesis nor the self-regulation of living bodies can be explained in terms of material structure alone, and he writes :—

"Consider the autogenesis of a particular moth from the egg. The egg is a minute speck of protoplasm containing in a semi-fluid matrix a number of distinguishable microscopic particles of genes, arranged in a fairly definite pattern ; and no doubt each of these particles may have a definite material structure or pattern of arrangement of its constituent elements, whatever these may be—and there is good evidence that each gene is somehow necessary to the development of one or more of the features of the adult organism. The egg cell divides again and again, masses of cells become differentiated to form special organs, each vastly complex and exactly fitted to play its part in the economy of the whole : and the whole assemblage of organs, which is the caterpillar, displays complex instincts. Then a process of resolution sets in. The organs are resolved into a mass of cells within a bag of skin, a mass that seems little more than a drop of pus. Then a new organizing process begins—new organs gradually form, all different from those of the caterpillar, many of them entirely new and complex; and then the moth emerges to display a new and entirely different array of complex instincts."

Then, again, in connection with the self-regulation of living bodies, what force is at work which sets in operation the formation of antimicrobic and antitoxic substances? If the body could, when invaded by microbes, elaborate some general antimicrobic serum it would be a marvel. But that it does in many cases produce an antimicrobic substance which is specific to a given microbe and only to that one is, to say the least, extraordinary; more particularly when we remember that it may never have encountered that particular microbe before. The same with antitoxic substance; some most complicated chemical toxin gains a foothold in the body, and some force sets to work to produce a most complex chemical substance which has a perfect neutralizing effect on that particular toxin. What is the force which does this, and what is its main characteristic, if it is not purpose?

Professor Francis tells me that the study of the changes produced in organic chemical substances by their passage through the animal organism has led to the generalization that such changes tend to the formation of less toxic bodies, and has supplied me with many examples. This again is a very remarkable fact, when we bear in mind that the body has had no previous experience of such substances, and such a substance may be an entirely new synthetic product, which may never have existed before.

Many events are so common and so perpetually seen that we take them as a matter of course, and hear little or no speculation as to why or how they occur. I refer to such things as the healing of a wound, and the extraordinary events which take place in the mending of a bony fracture. Such events would not have occurred had there been no wound or no fracture, and we must admit that they are purposive in character, *i.e.* teleological functions which mould structure according to their will.

As in the body we see these purposive functions at work, so in the mind are the main functions teleological. No one knows what life is, though each of us has some acquaintance with it; and just as the properties of iron filings in a magnetic field could not be deduced merely by examination of the filings, so, probably, the properties of the chemical components of the body cannot be understood without postulating some unknown factor outside them which may be called a "life field."

A very brief allusion must be made to telepathy, a condition in which almost certainly there is a direct influence of mind on mind, irrespective of distance or space. This phenomenon appears to depend on intensity of interest, or great sympathy between the persons concerned. The mental activity of one person may therefore be influenced by the mental activity of another without the intervention of any known physical structure. If this be so, we must be very chary before we insist on any causal relation of cell to mind in the brain. We must beware of being blind to the processes of actual life, while we are carried away by our observations of microscopic structure. The only way in which we can escape from the acceptance of telepathic phenomena is to disbelieve the careful and critical evidence which has been collected and impartially sifted by some of the most competent investigators.

To sum up, the body wants to do something, and so it produces a structure by means of which it may carry out its wish. The whole process is a purposive and teleological one. This is its striving, and to attain its end it uses many and diverse methods.

In this address I have tried to show that function implies a striving towards some end, and to attain its goal it develops and modifies structure. The structure so obtained is the substance through which it is manifested. Does it not follow that if functions are energetically and persistently used through a long series of generations the suitable pathways for the exhibition of those functions will be proportionately free or facilitated in the offspring ? Further, if this be so, may not modern legislation which removes personal responsibility, and diminishes individual striving, be a profound biological fault, leading to racial deterioration ?

I have quoted the views of biologists, physiologists, anatomists, neurologists, psychologists, chemists and physicists. I will end by a quotation from the late Poet Laureate, a philosopher, poet and doctor :—

"Thus must all kind of stimulus hav come some way
across the misty march-land, whereon men would fix
their disputable boundary between Matter and Mind,
—as every sensation must suffer translation
ere it can mediate in the live machinery
of any final cause or purpose : whence 'twould seem
that science went astray thinking to appropriate
some nervous reactions wholly to her material sphere,
and rather should have thought to extend the mental field."

Robert Bridges, "The Testament of Beauty."

The writer acknowledges his indebtedness to the work of Wood Jones and Porteus on "The Development of the Nervous System" in *The Matrix of the*

*Mind*, from which Figs. 2, 3 and 4 are taken, and also to *Modern Materialism*, by W. McDougall, both of which he has used freely. Among other books of reference are :—

J. S. Haldane, *The Philosophical Basis of Biology.*

A. S. Eddington, *The Nature of the Physical World*

C. D. Broad, *Mind and its Place in Nature.*

J. H. Woodger, *Biological Principles.*

J. S. Collier, The Savill Memorial Lecture, 1929.

## Figures and Tables

**Fig. 1. f1:**
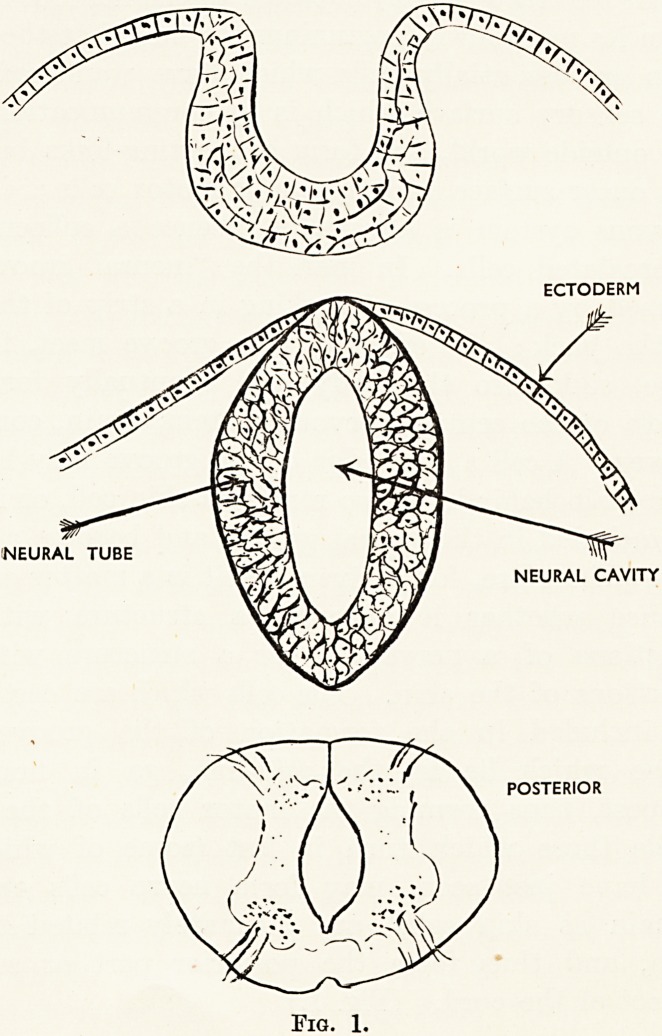


**Fig. 2. f2:**
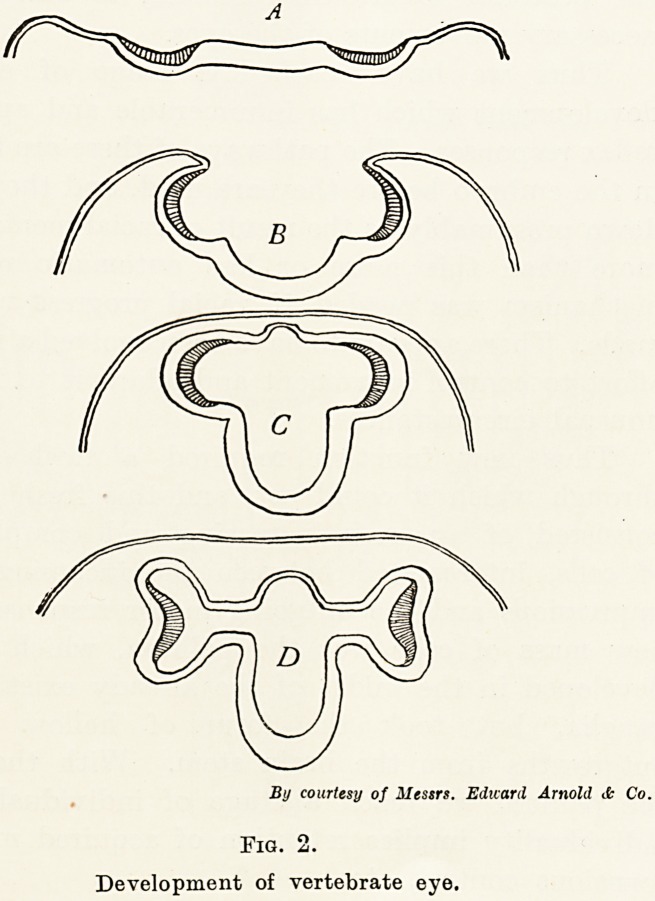


**Fig. 3. f3:**
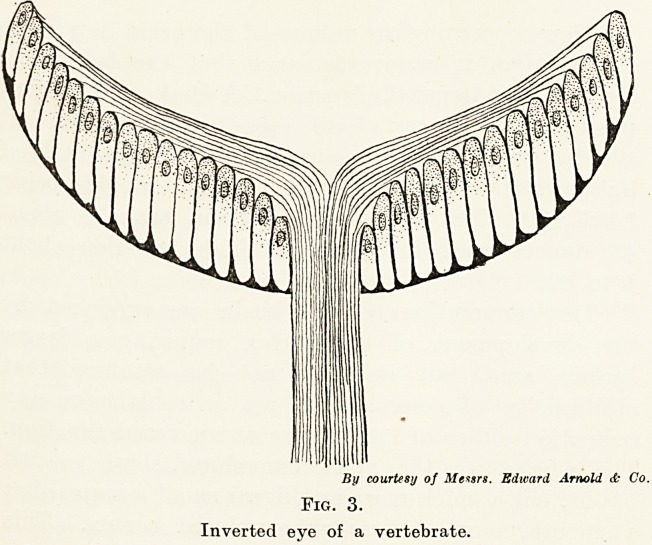


**Fig. 4. f4:**
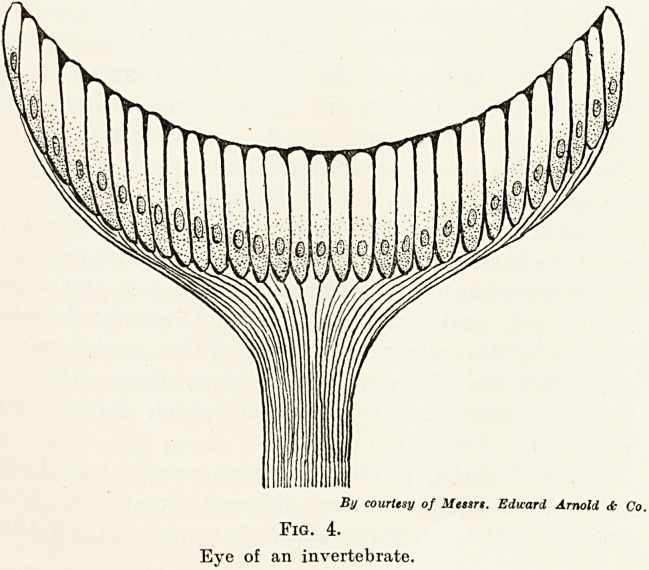


**Fig. 5. f5:**
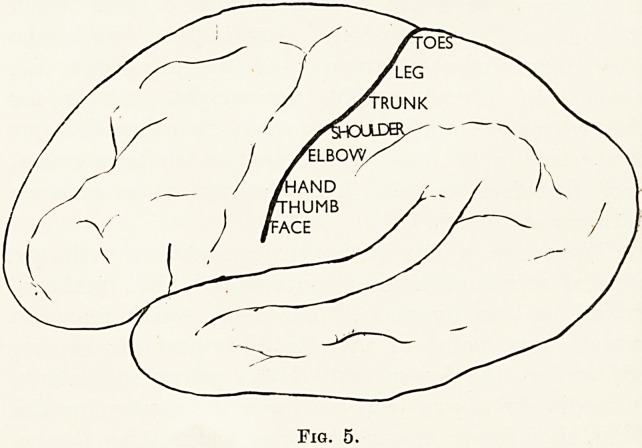


**Fig. 6. f6:**
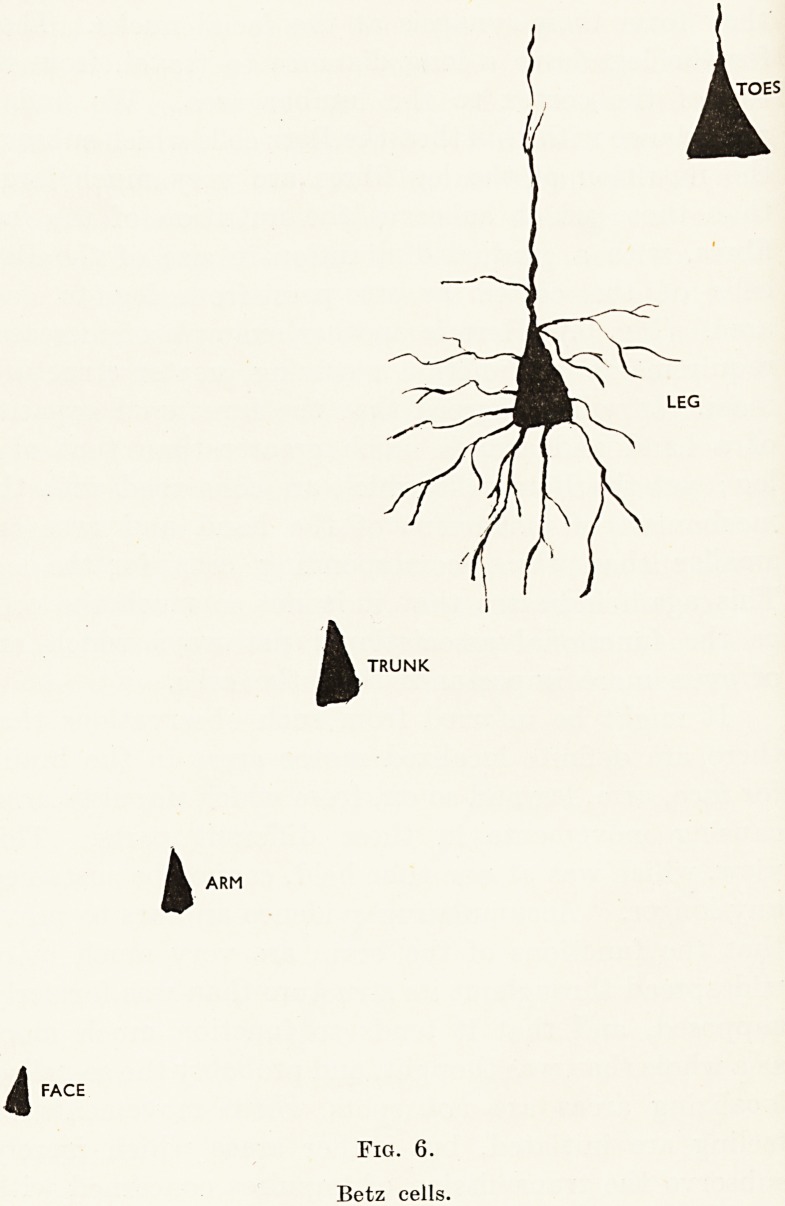


**Fig. 7. f7:**